# Using mixture models to characterize disease-related traits

**DOI:** 10.1186/1471-2156-6-S1-S99

**Published:** 2005-12-30

**Authors:** Tao Duan, Stephen J Finch, Kenny Q Ye, Gary A Chase, Nancy R Mendell

**Affiliations:** 1 Stony Brook University, Stony Brook, NY, 11794, USA; 2 Albert Einstein College of Medicine, Bronx, NY, 10461, USA; 3 The Pennsylvania State University College of Medicine, Hershey Medical Center, 600 Centerview Drive, Box855, Hershey, PA 17033, USA

## Abstract

We consider 12 event-related potentials and one electroencephalogram measure as disease-related traits to compare alcohol-dependent individuals (cases) to unaffected individuals (controls). We use two approaches: 1) two-way analysis of variance (with sex and alcohol dependency as the factors), and 2) likelihood ratio tests comparing sex adjusted values of cases to controls assuming that within each group the trait has a 2 (or 3) component normal mixture distribution. In the second approach, we test the null hypothesis that the parameters of the mixtures are equal for the cases and controls. Based on the two-way analysis of variance, we find 1) males have significantly (*p *< 0.05) lower mean response values than females for 7 of these traits. 2) Alcohol-dependent cases have significantly lower mean response than controls for 3 traits. The mixture analysis of sex-adjusted values of 1 of these traits, the event-related potential obtained at the parietal midline channel (ttth4), found the appearance of a 3-component normal mixture in cases and controls. The mixtures differed in that the cases had significantly lower mean values than controls and significantly different mixing proportions in 2 of the 3 components. Implications of this study are: 1) Sex needs to be taken into account when studying risk factors for alcohol dependency to prevent finding a spurious association between alcohol dependency and the risk factor. 2) Mixture analysis indicates that for the event-related potential "ttth4", the difference observed reflects strong evidence of heterogeneity of response in both the cases and controls.

## Background

Disease-related traits (DRTs) may provide more powerful phenotypes than the disease itself for identifying alcohol dependency genes. For example, an alcohol DRT phenotype might be due to a single major gene with high penetrance, while alcohol dependency may result from the action of several genes and environmental factors. In characterizing a DRT, we first compare affected to unaffected individuals. If the DRT is due to 1 of many disease predisposing genes, then the responses in both affected and unaffected individuals may be a mixture with 2 or 3 components depending on the effect of genotype on the DRT in affected individuals and unaffected individuals. Lo et al. [1] successfully applied this idea in their study of working memory, a schizophrenia-related trait. Assuming a within-group mixture of an exponential and a normal distribution, they found significant differences between normal controls and relatives of patients with schizophrenia. These results had not been noted when they compared these two groups using traditional 2-sample tests comparing means or medians.

## Methods

### The sample

We considered Collaborative Study on the Genetics of Alcoholism (COGA) family data provided by Genetic Analysis Workshop 14 (GAW14) Problem 1 [2]. One affected individual was sampled at random from each of the 105 families providing data on the electrophysiological measures. We then randomly sampled one "purely unaffected" individual from those families, when such a person was available. The result was a sample of 105 cases, the alcohol-dependent affected individuals, and 50 controls, the purely unaffected individuals. Seventy-three percent of the affected individuals were male, and 22% of the unaffected individuals were male.

### Variables

The 12 event-related potentials (ERPs), phenotypes ttth1, ttth2, ttth3, ttth4, ttdt1, ttdt2, ttdt3, ttdt4, ntth1, ntth2, ntth3, and ntth4, and one electroencephalogram (EEG) phenotype, ecb21, were considered, as well as the sex of the individual.

### Statistical methods

The 2-way analysis of variance used sex, disease status (affected vs. unaffected), and the sex-disease status interaction as factors.

A mixture model analysis incorporated the findings on sex obtained in the 2-way analysis of variance, and was done on sex-adjusted values for those traits in which we found a difference between males and females. The adjustment was Y_Adj _= Y - d_f_, where  for females and d_f _= 0 for males.

This analysis assumed that conditional on whether an individual is a case or control, the distribution of the trait has a 2-component normal mixture distribution. If we let *X *= 1 when an individual is a control and *X *= 2 when an individual is a case, then the density of the trait, *Y*, is

*f*_*x*_(y) = π_1*x *_φ(*y*; μ_1*x*_, σ) + π_2*x *_φ(*y*; μ_2*x*_, σ) for *x *= 1, 2,     (1)

where φ(*y*; μ, σ) denotes the normal density with mean, μ, and standard deviation, σ and π_1*x *_+ π_2*x *_= 1. Without loss of generality, μ_1*x *_< μ_2*x *_and 0 < π_*ix *_< 1 for *x *= 1, 2.

The null hypothesis

H_00_: μ_*i*2 _= μ_*i*1 _*and *π_*i*2 _= π_*i*1 _for *i *= 1, 2     (2)

can be tested against the alternative of equal component means and unequal mixing proportions

H_01_: μ_*i*2 _= μ_*i*1 _and π_*i*2 _≠ π_*i*1 _for *i *= 1, 2     (3)

using a likelihood ratio test (LRT) statistic. Under the null hypothesis, the LRT statistic has an asymptotic chi-square distribution with 1 df. We can also consider an alternative of unequal means and equal mixing proportion, i.e.,

H_10_: μ_*i*2 _≠ μ_*i*1 _and π_*i*2 _= π_*i*1 _for *i *= 1, 2.     (4)

Finally we also consider an alternative of unequal means and unequal mixing proportions

H_11_: μ_*i*2 _≠ μ_*i*1 _and π_*i*2 _≠ π_*i*1 _for *i *= 1, 2.     (5)

If we reject H_00 _we might want to consider the alternative H_11 _given in (5) versus H_01 _using a 2 df chi-square test or H_10 _using a 1 df chi square test.

Following the same logic, we considered a set of 3-component normal mixture models for cases and controls. Similar to model (1), we considered 3 component mixtures with equal within component variances. Thus the general equation for the mixture density is

*f*_*x*_(*y*) = π_1*x *_φ(*y*; μ_1*x*_, σ) + π_2*x *_φ(*y*; μ_2*x*_, σ) + π_3*x *_φ(*y*; μ_3*x*_, σ) for *x *= 1, 2,     (6)

where π_1*x *_+ π_2*x *_+ π_3*x *_= 1 and 0 < π_*ix *_< 1 for *x *= 1, 2, *i *= 1, 2, 3. We again set μ_1*x *_< μ_2*x *_< μ_3*x*_, and refer to the component having mean μ_*ix *_as the *i*^th ^component. As in comparing cases to controls assuming a 2-component normal mixtures, we estimate the parameters and test hypotheses under various 3-component normal mixture models. These include 1) equal parameter values for cases and controls (6 parameters); 2) unequal mixing proportions, but equal component means (8 parameters); 3) unequal component means but equal mixing proportions (9 parameters); 4) equal first component means and equal first component mixing proportions (9 parameters); 5) unequal mixing proportions and unequal within component means (11 parameters).

The expectation-maximization algorithm (EM) [3], a general approach to maximum likelihood estimation (MLE), is applied to estimate parameters π_*ix*_, μ_*ix *_*and *σ for *i *= 1, 2 (or 3) and *x *= 1, 2.

A method of Maller and Zhou [4] allows us to test specific hypotheses using the LRT. However, when the mixing proportions are on the boundary of the parameter space and the parameters are not identifiable under the null model, the LRT does not follow the usual asymptotic chi-square distribution with degrees of freedom equal to the difference in the number of parameters between the 2 hypotheses. In this case, to select the model, we considered both the Akaike information criterion (AIC) [5] and the Bayesian information criterion (BIC) [6]. In using AIC and BIC, we selected the model with the smallest AIC or BIC value. We used both because in many model selection studies, it is found that AIC tends to select more complex models, while BIC tends to penalize complex models heavily, giving preference to simpler models. This appears to hold in selecting the number of components in the mixture analysis [7].

## Results

### 2-way analysis of variance

Table 1 shows the results of 2-way analysis of variance. In each of the 13 DRTs, the sex-disease interaction was nonsignificant (all *p *> 0.17). For 7 traits, sex was a significant factor; disease was a significant factor for only 3 traits. This was unexpected, because based on the data description, we expected to observe differences between cases and controls on all measures. In Table 1 we report the confidence intervals for the means on comparing cases to controls and on comparing males to females.

### Mixture analysis

The means observed for males were slightly lower than those observed for females wherever there was a significant sex difference. Thus the adjusted values for females were slightly smaller than the original values. The values of the adjustment used in the females for the electrophysiological measures, when there was a significant sex difference, range from 0.32 to 2.00, with the sex adjustment value for the ERP obtained at the parietal midline channel, ttth4, equal to 0.45.

We failed to reject H_00 _for the alternative H_01 _(equal means, unequal mixing proportions) for every DRT considered. We rejected H_00 _at the 0.05 level for alternative H_10 _(equal mixing proportions, unequal means) in the case of trait ttth2 (χ^2 ^= 6.3, df = 2, *p*-value = 0.04). In the case of ttth4, the DRT obtained at the parietal midline channel, we reject H_00 _(χ^2 ^= 8.9, df = 3, *p*-value = 0.03), H_01 _(χ^2 ^= 7.1, df = 2, *p*-value = 0.03), and H_10 _(χ^2 ^= 4.4, df = 1, *p*-value = 0.04) for H_11_, indicating that we may have both unequal mixing proportions and unequal means for cases and controls. Thus, while the analysis of variance shows that the controls have a higher mean value of ttth4, the mixture analysis indicates more complex distribution differences. Both component means are higher in the controls than the cases (3.83 vs. 3.33 and 6.00 vs. 4.64), whereas the estimated proportion of controls in component with the higher mean is lower than that for the cases (0.04 vs. 0.31).

Applying similar methods we used LRTs to find the most parsimonious 3-component normal mixture distribution for ttth4. Upon doing this we rejected hypotheses of equal mixing proportions for cases and controls (χ^2 ^= 12.6, df = 2, *p*-value = 0.002) and of equal component means for cases and controls (χ^2 ^= 13.4, df = 3, *p*-value = 0.004). Upon exploring further, we could not reject a hypothesis that cases and controls had equal means and proportions in the first component, i.e., the component with the lowest mean (χ^2 ^= 0.2, df = 2, *p*-value > 0.9).

**Figure 1:**
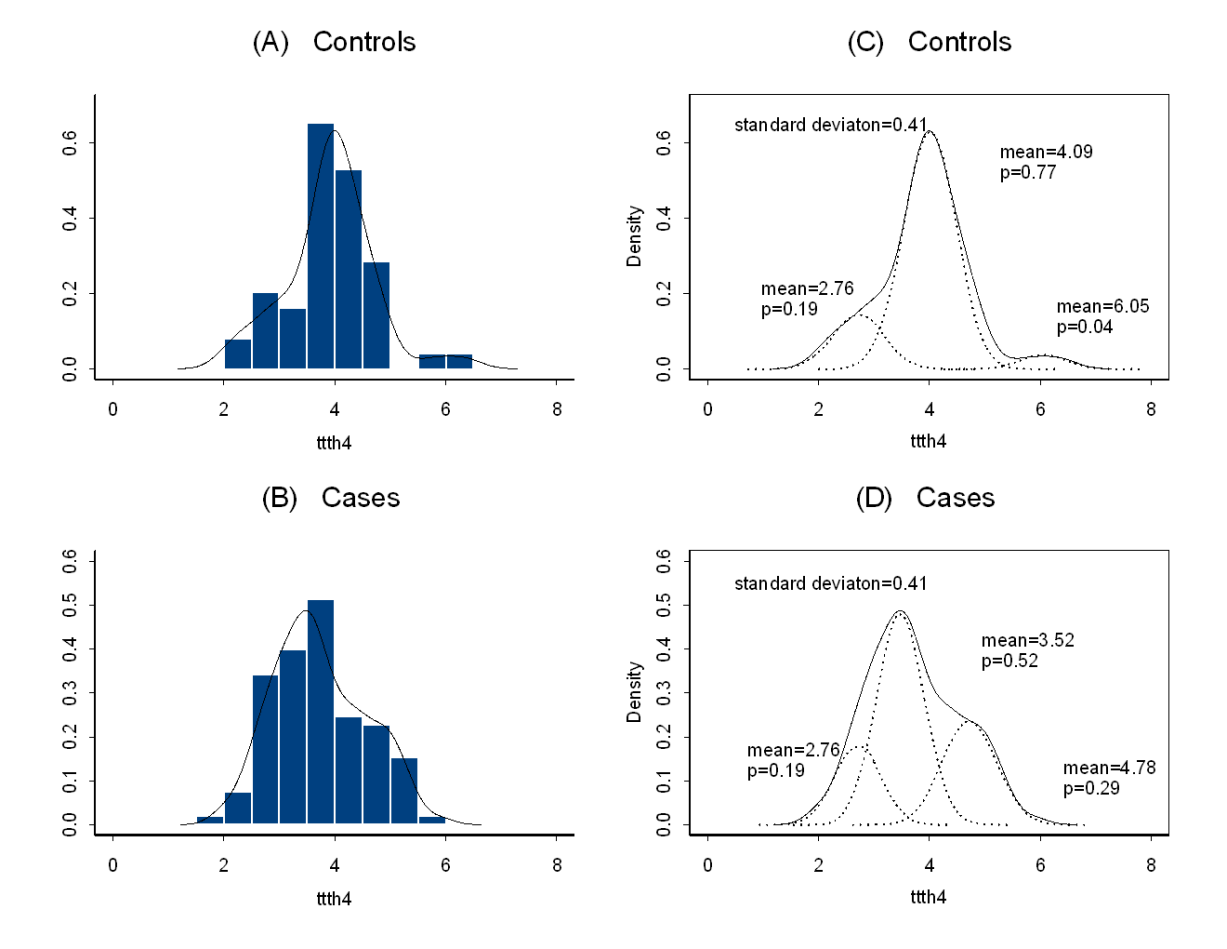
Density polygons for alcohol-related trait ttth4: cases vs. controls.

Using AIC and BIC, we compared the likelihoods of our most parsimonious models accounting for the differences in cases and controls. That is, we compared the likelihoods of a 1-component normal density model (with cases and controls having unequal means and equal variances), to a 2-component normal mixture model (with cases and controls having unequal mixing proportions and unequal component means), and to a 3-component normal mixture model (with cases and controls having unequal mixing proportions and unequal means for 2 out of 3 of the components). The AIC values of the single normal density, 2-component mixture model and 3-component mixture model are 381.2, 380.8, and 371.5, respectively. The BIC values for the above three models are 390.3, 402.1, and 398.8, respectively. AIC leads to a 3-component mixture model, while a single density model is indicated by BIC. When there are inconsistencies in model selection based on AIC and BIC, Leroux [8] recommends the choice of the number of components might be based on a direct comparison of the fitted frequency distributions. Figure 1(A, B) contains the density histograms in cases and controls for this trait, ttth4. It shows that a single normal density does not appear to be sufficient. Based on this, we have selected the 3-component mixture model as most appropriate. Thus our selected model for the distribution of ttth4 is

*f*_1_(y) = 0.19 φ(*y*; 2.76, 0.41) + 0.77 φ(*y*; 4.09, 0.41) + 0.04 φ(*y*; 6.05, 0.41) for controls

and

*f*_2_(y) = 0.19 φ(*y*; 2.76, 0.41) + 0.52 φ(*y*; 3.52, 0.41) + 0.29 φ(*y*; 4.78, 0.41) for cases.

Figure 1(C, D) plots these mixtures.

## Discussion

In the case of ttth4, the first component mean and corresponding mixing proportion are the same for cases and controls, and there is a general shift, in the direction that the mean ttth4 is lower for alcohol-dependent individuals than their unaffected relatives in the other 2 component means. From the final model, we can see that the explanation for a lower mean in the cases is the lower mean and a lower estimated proportion in the second component compared to the control group.

An interesting result is that, with sex controlled, there are few significant differences between cases and controls, namely ttth4, ttdt3, and ttdt4. For moderate estimated effect size , with a sample size *n *= 50 in each group, power is equal to or larger than 0.50. Thus we have reasonable power to detect differences between cases and controls. Whenever our alcohol-dependent sample has a larger percentage of males than our control sample, any differences observed between cases and controls may reflect these sex differences rather than differences in the disease groups. Regardless of the sample makeup, taking sex into account should always be done when studying factors related to alcoholism. Another reason we do not see large differences between our controls and the cases may be that these controls all have a family history of alcoholism.

In this study we report significant findings observed on investigating 13 correlated measures. As in any study in which a large number of tests have been done, we would expect some significant findings due to chance. Thus the results here must be considered as preliminary. On the other hand, given that these measures were included in the COGA dataset [2] as being potential alcohol risk factors, it is rather surprising that so *few *significant findings are observed on comparing cases to controls.

## Conclusion

Two-way analysis of variance (sex and disease) indicates that controlling for sex there is a significant difference between alcohol-dependent cases and controls for only 3 ERPs, namely ttth4, ttdt3, and ttdt4. Comparison of both the 2-component and 3-component normal mixture parameters for ttth4, the ERP obtained at the parietal midline channel, indicate these differences may reflect the same mixing proportion and mean in the component having the lowest mean, but unequal mixing proportions and unequal component means in the other 2 components.

## Abbreviations

AIC: Akaike information criterion

BIC: Bayesian information criterion

COGA: Collaborative Study on the Genetics of Alcoholism

DRT: Disease-related trait

EEG: Electroencephalogram

EM: Expectation-maximization algorithm

ERPs: Event-related potentials

GAW14: Genetic Analysis Workshop 14

LRT: Likelihood ratio test

MLE: Maximum likelihood estimation

## Authors' contributions

NRM, SJF, KQY, and GAC conceived of the study, participated in its design and coordination, and helped to draft the manuscript. NRM presented this work. TD carried out all of the analyses including the genetic analyses, data reduction, and statistical analyses.

## Figures and Tables

**Table 1:** Two-way ANOVA of disease-related traits in probands and siblings

	Confidence interval (mean ± SE)				*p*-Value	
		
Trait	Case (*n *= 105)	Control (*n *= 50)	Male (*n *= 88)	Female (*n *= 67)	Disease	Sex
ttth1	2.47 ± 0.07	2.42 ± 0.10	2.44 ± 0.07	2.47 ± 0.08	0.71	0.62
ttth2	4.01 ± 0.10	4.20 ± 0.14	3.97 ± 0.11	4.20 ± 0.12	0.27	0.29
ttth3	4.22 ± 0.10	4.56 ± 0.14	4.12 ± 0.11	4.61 ± 0.12	0.06	0.02^a^
ttth4	3.85 ± 0.08	4.26 ± 0.12	3.79 ± 0.09	4.24 ± 0.10	0.01^b^	0.03^a^
ttdt1	2.98 ± 0.09	2.71 ± 0.13	2.88 ± 0.09	2.93 ± 0.11	0.08	0.17
ttdt2	3.64 ± 0.10	3.85 ± 0.14	3.44 ± 0.10	4.06 ± 0.12	0.20	0.00^b^
ttdt3	4.10 ± 0.10	4.49 ± 0.14	3.85 ± 0.11	4.71 ± 0.12	0.03^a^	0.00^b^
ttdt4	4.51 ± 0.12	5.07 ± 0.17	4.39 ± 0.13	5.08 ± 0.15	0.01^b^	0.02^a^
ntth1	1.90 ± 0.05	1.84 ± 0.07	1.88 ± 0.05	1.87 ± 0.06	0.52	0.82
ntth2	2.78 ± 0.07	2.81 ± 0.10	2.70 ± 0.08	2.89 ± 0.09	0.81	0.10
ntth3	2.99 ± 0.07	3.08 ± 0.10	2.84 ± 0.08	3.26 ± 0.09	0.50	0.00^b^
ntth4	3.00 ± 0.07	3.12 ± 0.10	2.90 ± 0.08	3.22 ± 0.09	0.33	0.01^b^
ecb21	13.87 ± 0.53	15.17 ± 0.79	13.43 ± 0.58	15.42 ± 0.68	0.17	0.07

^a ^Significant at 0.05

^b ^Significant at 0.01
